# Predictive Factors for RAI-Refractory Disease and Short Overall Survival in PDTC

**DOI:** 10.3390/cancers13071728

**Published:** 2021-04-06

**Authors:** David Kersting, Robert Seifert, Lukas Kessler, Ken Herrmann, Sarah Theurer, Tim Brandenburg, Henning Dralle, Frank Weber, Lale Umutlu, Dagmar Führer-Sakel, Rainer Görges, Christoph Rischpler, Manuel Weber

**Affiliations:** 1 Department of Nuclear Medicine, University Hospital Essen, University Duisburg-Essen, Hufelandstraße 55, 45147 Essen, Germany; david.kersting@uk-essen.de (D.K.); robert.seifert@uk-essen.de (R.S.); lukas.kessler@uk-essen.de (L.K.); ken.herrmann@uk-essen.de (K.H.); Rainer.Goerges@uk-essen.de (R.G.); christoph.rischpler@uk-essen.de (C.R.); 2 Institute of Pathology, University Hospital Essen, University Duisburg-Essen, Hufelandstraße 55, 45147 Essen, Germany; sarah.theurer@uk-essen.de; 3 Department of Endocrinology and Metabolism, Division of Laboratory Research, University Hospital Essen, University Duisburg-Essen, Hufelandstraße 55, 45147 Essen, Germany; tim.brandenburg@uk-essen.de (T.B.); Dagmar.Fuehrer-Sakel@uk-essen.de (D.F.-S.); 4 Department of General, Visceral and Transplantation Surgery, Section of Endocrine Surgery, University of Duisburg-Essen, Hufelandstraße 55, 45147 Essen, Germany; henning.dralle@uk-essen.de (H.D.); frank.weber@uk-essen.de (F.W.); 5 Department of Diagnostic and Interventional Radiology and Neuroradiology, University Hospital Essen, University Duisburg-Essen, Hufelandstraße 55, 45147 Essen, Germany; lale.umutlu@uk-essen.de

**Keywords:** poorly differentiated thyroid carcinoma, PDTC, radioactive iodine treatment, PET/CT, ^124^I, ^131^I, dual imaging

## Abstract

**Simple Summary:**

Poorly differentiated thyroid cancer is a rare subtype of thyroid cancer. The course of this disease can vary substantially. Treatment options consist of surgery and radioactive iodine therapy, if possible, and in tyrosine kinase inhibitors for patients where this is not possible. The aim of this study was to identify risk factors for the development of disease that does not respond to radioactive iodine therapy and for premature death, in order to better identify patients in need of more extensive tumor staging and treatment. We identified primary tumor size and infiltration of the tissue surrounding the thyroid gland as risk factors for the development of disease that does not respond to radioactive iodine therapy and tumor volume as a risk factor for early death.

**Abstract:**

Background: The clinical phenotype of poorly differentiated thyroid cancer (PDTC) can vary substantially. We aim to evaluate risk factors for radioiodine refractory (RAI-R) disease and reduced overall survival (OS). Methods: We retrospectively screened our institutional database for PDTC patients. For the assessment of RAI-R disease, we included patients who underwent dual imaging with ^18^F-FDG-PET and ^124^I-PET/^131^I scintigraphy that met the internal standard of care. We tested primary size, extrathyroidal extension (ETE), and age >55 years as risk factors for RAI-R disease at initial diagnosis and during the disease course using uni- and multivariate analyses. We tested metabolic tumor volume (MTV), total lesion glycolysis (TLG) on ^18^F-FDG-PET, and the progression of stimulated thyroglobulin within 4–6 months of initial radioiodine therapy as prognostic markers for OS. Results: Size of primary >40 mm and ETE were significant predictors of RAI-R disease in the course of disease in univariate (81% vs. 27%, *p* = 0.001; 89% vs. 33%, *p* < 0.001) and multivariate analyses. Primary tumor size was an excellent predictor of RAI-R disease (AUC = 0.90). TLG/MTV > upper quartile and early thyroglobulin progression were significantly associated with shorter median OS (29.0 months vs. 56.9 months, *p* < 0.05; 57.8 months vs. not reached *p* < 0.005, respectively). Discussion: PDTC patients, especially those with additional risk factors, should be assessed for RAI-R disease at initial diagnosis and in the course of disease, allowing for early implementation of multimodal treatment. Primary tumor size >40 mm, ETE, and age >55 are significant risk factors for RAI-R disease. High MTV/TLG is a significant risk factor for premature death and can help identify patients requiring intervention.

## 1. Introduction

Poorly differentiated thyroid carcinoma (PDTC) is a rare subtype of thyroid carcinoma representing about 2–3% of thyroid cancers (TC) in the United States [1]. PDTCs show an intermediate level of differentiation when compared to anaplastic thyroid carcinoma on one side and papillary or follicular thyroid carcinoma on the other side of the spectrum [1]. Five-year survival rates range from approximately 65 to 85% and can vary substantially [2,3,4]. PDTC lesions are at an increased risk of being radioiodine refractory (RAI-R) at initial diagnosis or becoming so in the course of disease [5,6]. Subsequently, the benefit of radioactive iodine therapy (RAIT) in these patients is variable and the routine treatment with RAIT is therefore largely controversial. However, patients with PDTC should not be automatically precluded from undergoing RAIT, as RAI− avid disease is described in about 25% of patients [7]. It is therefore crucial to reliably identify patients likely to benefit from RAIT as well as patients with RAI-R TC or are at a high risk for progression to RAI-R TC. Given the toxicity profile of current RAI-R TC medications, such as hypertension, skin reactions and proteinuria [8,9] it is of additional importance to then reliably stratify patients into those at increased risk for cancer-related death and in need for therapeutic intervention vs. those that can be managed with active surveillance [10].

As metabolic tumor volume (MTV) and total lesion glycolysis (TLG) are derived from ^18^F-FDG PET and have shown to be inversely correlated with progression-free survival and overall survival (OS) in different tumor entities, among which is RAI-R TC [11]. We hypothesize that it can aide risk stratification in patients with PDTC.

The aims of this study were to determine predictive factors for RAI-R TC in PDTC at initial diagnosis and in the course of disease, as well as to establish parameters that identify patients at a high risk for early mortality.

## 2. Methods

We retrospectively screened our institutional database for all patients treated at our hospital from 2007 until March 2020 meeting the following inclusion criteria:

Histopathologically confirmed poorly differentiated thyroid cancer (PDTC) in accordance with the Turin proposal [2]. Patients with an earlier initial diagnosis were considered if a re-evaluation of the original histopathology had been performed after the publication of the Turin proposal.

For the assessment of RAI-R TC: Dual imaging, defined as ^18^F-FDG PET and ^124^I-PET *or* ^131^I whole-body scan (WBS) meeting the internal standard of care for PDTC.

Patients with ^18^F-FDG PET but without iodine imaging were included for the analysis of MTV and TLG as prognostic factors for OS.

### 2.1. RAI Avidity

Based on initial dual imaging, patients were classified as RAI−avid (RAI+), RAI-Refractory (RAI−), and disease-free (DF). RAI-Refractory disease was defined as the absence of RAI-uptake in at least one lesion or radiological disease progression within one year after RAIT. For the assessment of RAI-R in the course of disease, structural persistence after the administration of cumulatively 22.0 GBq ^131^I was added as a criterion. We tested primary tumor size >40 mm, any extrathyroidal extension, and age > 55 years as putative risk factors for RAI-R disease at initial diagnosis and in the course of disease based on the evidence for their prognostic role in TC [12].

### 2.2. Response Evaluation

Treatment response was assessed one year after initial treatment and classified as either an excellent response, a biochemical incomplete response, a structural incomplete response, or an indeterminate response in adherence to current ATA guidelines [13]. Time to progression to RAI-R TC was defined as time from initial RAIT until at least one of the abovementioned criteria was met.

### 2.3. Predictive Factors for Overall Survival

Early thyroglobulin (Tg) progression, FDG- avid disease, MTV, TLG, and TNM stage were tested as prognostic factors for overall survival. Early Tg progression was defined as any increase in stimulated Tg at 4–6 months after initial treatment. FDG-avid disease was defined as the presence of any focal FDG-uptake unambiguously identified as neoplastic using both PET and CT information. AJCC TNM stage was stratified in accordance with the 7th edition.

MTV and TLG were obtained from ^18^F-FDG-PET using a research software prototype (MIWBAS, version 1.0, Siemens Medical Solutions USA, Inc., Knoxville, TN, USA). A local threshold of 50% of SUVmax was used. OS was defined as time from initial RAIT to death/last follow-up when analyzing early Tg progression and the time from FDG-PET/CT to death/last follow-up when analyzing MTV and TLG.

### 2.4. Statistical Analysis

Statistical analysis was performed using IBM SPSS Statistics for Mac, version 26 (IBM Corp., Armonk, NY, USA). Interval data are given in the format mean ± standard deviation. Fisher’s exact test was used to determine statistical significance for differences among groups and logistic regression was used for multivariate analysis for the patients where tumor size and ETE were known. A *p*-value < 0.05 was considered statistically significant. A log-rank test using Kaplan-Meier curves was used to test assumed predictive factors for OS. Receiver operating characteristic (ROC) analysis was performed to assess the predictive value of primary tumor size for radioiodine refractoriness using area under the curve (AUC) as a metric. Youden’s J statistics were employed to identify the optimal cutoff value for primary tumor size and their association with radioiodine refractoriness.

## 3. Results

### 3.1. Imaging and Treatment Protocol

Initial RAIT was carried out on clinical indication in 47/51 patients after the oral administration of 3.9 ± 2.5 Gbq ^131^I. Prior dosimetry using ^124^I PET was employed to assess RAI avidity before initial and follow-up RAIT, if the likelihood for insufficient RAI uptake was deemed significant. Based on prior publications [14,15,16], in the presence of iodine-avid lesions on pretherapeutic ^124^I PET imaging, dosimetry-based RAIT activities aiming at tumor absorbed doses >85 Gy, while not exceeding a blood dose of 2.0 Gy, were administered. In the remainder, empirical activities were used.

On average, ^124^I PET was acquired 1 and 5 days after the oral administration of 26.2 ± 3.5 MBq ^124^I. ^18^F-FDG PET was performed 61.7 ± 9.1 min after the intravenous administration of 282.4 ± 65.2 MBq ^18^F-FDG. Mean interval between thyroidectomy and ^18^F-FDG PET was 4 months.

### 3.2. Study Cohort

Fifty-one patients (23 male, 28 female) were eligible for the assessment of RAI-R TC at initial diagnosis and in the course of disease. The mean patient age was 58.5 ± 17.3 years, and 30 (59%) patients were older than 55 years. In 2 patients the primary could not be evaluated (Tx) and the histopathological diagnosis was derived from metastatic tissue and in another 3, information about size of the primary tumor could not be retrieved. Thirty-one patients had a primary tumor >40 mm, and any extrathyroidal extension tumor was present in 28.

For the analysis of MTV and TLG as risk factors for OS, 4 patients had to be excluded, because the images were inaccessible, and two because of concurrent malignancies. An additional 11 patients that were referred to us for imaging only were included. These patients were not included in the assessment of RAI-R TC at initial diagnosis, as they were referred to our imaging center under the suspicion of RAI-R TC. Mean patient age of this cohort was 58.5 ± 18.0 years. Patient characteristics are provided in Table 1 and Table 2.

### 3.3. Initial RAI Avidity

**Figure 1:**
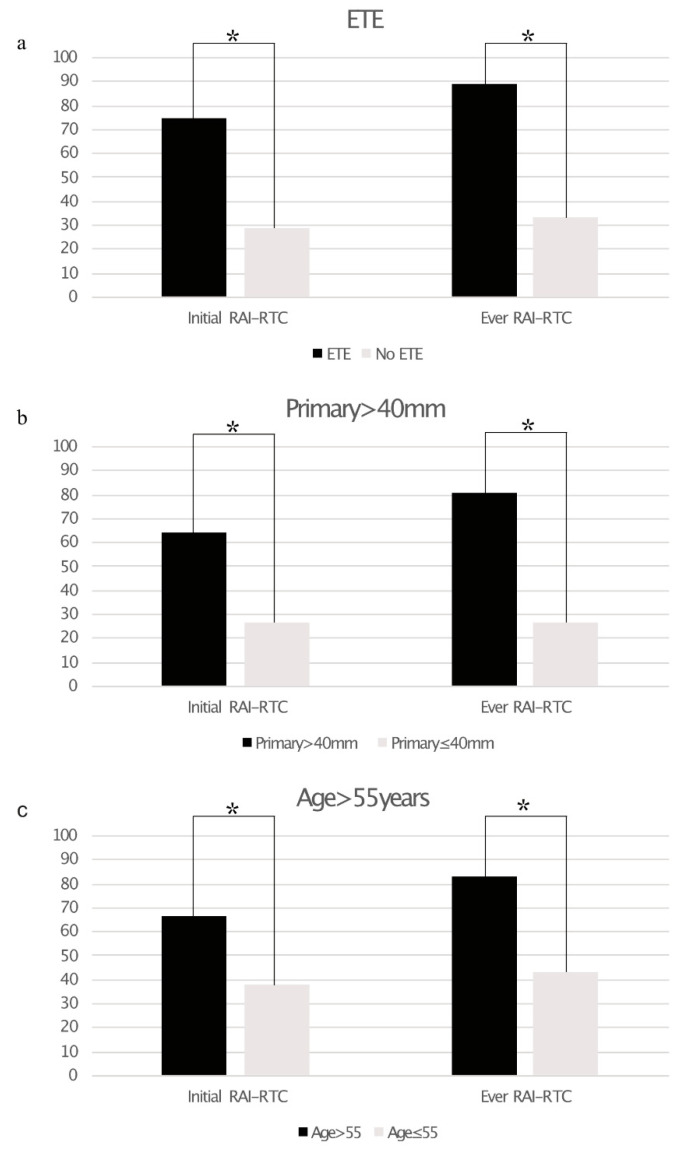
Bar graphs showing the prevalence of radioiodine refractory thyroid cancer (RAI-R TC) based on extrathyroidal extension ((**a**), ETE), size of primary tumor >40 mm (**b**), and age >55 years (**c**). Statistically significant differences are marked with an asterisk *.

A total of 19/51 (37%) of patients showed any neoplastic RAI uptake as assessed by ^124^I-PET alone (*n* = 4), ^131^I-WBS alone (*n* = 19) or both (*n* = 28). Post-therapeutic ^131^I-WBS detected additional lesions vs. ^124^I-PET in three patients. Using the aforementioned criteria, 16 patients were DF, 7 patients RAI+, and 28 patients RAI−. Of the latter 25 showed tumor lesions without iodine uptake at initial imaging and three patients developed radiological disease progression within 12 months of RAIT (mean: 8.0 ± 2.0 months). Patients with a primary tumor size >40 mm (relative risk 64.5% vs. 26.7%; *p* < 0.05), extrathyroidal tumor extension (75.0% vs. 28.6%; *p* < 0.005), and age >55 years (66.7% vs. 38.1%; *p* < 0.05) were significantly more likely to show RAI-R TC at initial diagnosis than patients without these risk factors. All of these risk factors were significantly associated with a higher risk for RAI-R TC in the course of disease (primary size >40 mm: 80.6% vs. 26.7%; *p* < 0.005, extrathyroidal extension: 89.3% vs. 33.3%; *p* < 0.001; age > 55 years: 83.3% vs. 42.9%, *p* < 0.005). Figure 1a–c and Table 3 gives an overview of the impact of the assessed risk factors on RAI-R TC.

**Figure 2:**
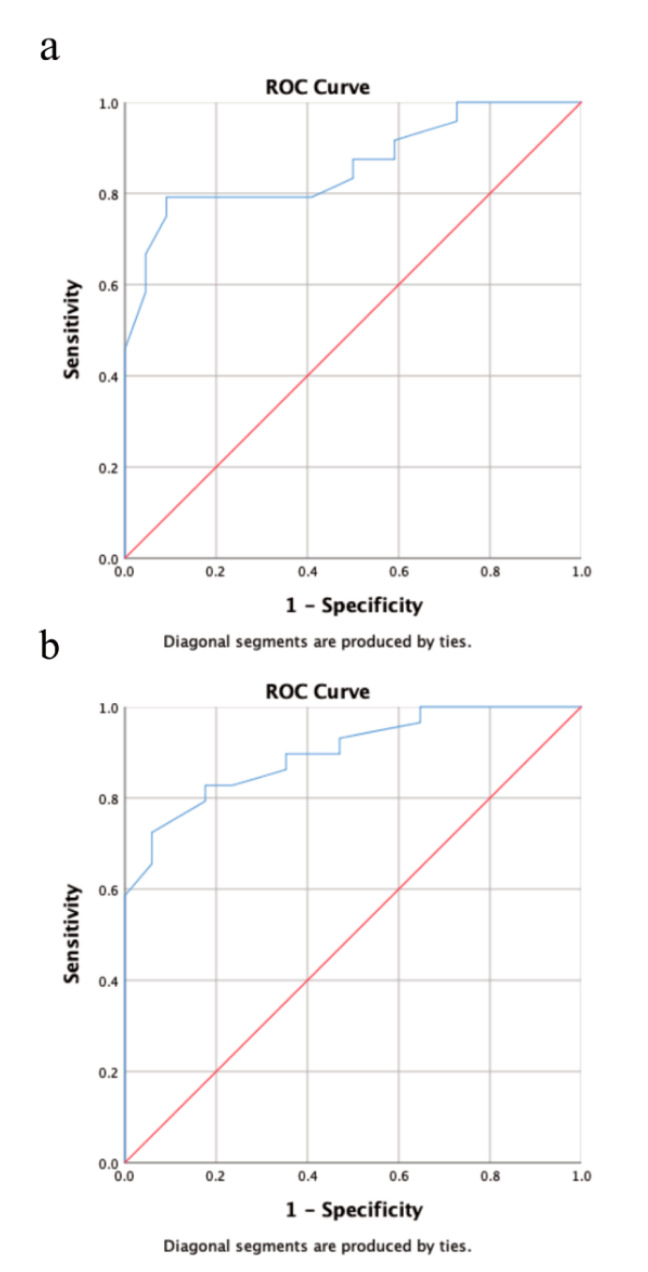
Receiver operating characteristic (ROC) analysis showing the predictive value for primary tumor size for RAI-R TC at initial diagnosis (**a**) and during the course of disease. (**b**) Using area under curve (AUC) as a metric primary tumor size was an excellent predictor for RAI-R TC at initial diagnosis (AUC = 0.86) and during the course of disease (AUC = 0.90).

Multivariate analysis revealed a statistically significant impact of extrathyroidal extension on RAI-R TC at initial diagnosis (*p* = 0.016; hazard ratio (95% confidence interval): 5.2 (1.3–19.8)), while primary tumor size >40 mm was barely not statistically significant (*p* = 0.08). Both factors were statistically significant for the onset of RAI-R TC in the course of disease (primary tumor size >40 mm: *p* = 0.007; hazard ratio (95% confidence interval): 11.6 (1.9–69.7); extrathyroidal extension: *p* = 0.003; 14.8 (2.5–87.0)). The results of the multivariate analysis are provided in Table 4. The AUC for the predictive value of primary tumor size was 0.86 for RAI-R TC at initial diagnosis and 0.9 for RAI-R TC in the course of disease. Youden’s J statistics identified a primary tumor size of 52.5 mm as the optimal cutoff value for the risk of RAI-R TC in the course of the disease (specificity: 94% sensitivity: 72.4%). The results of the ROC analysis are provided in Figure 2.

### 3.4. Therapy Response

A total of 14/16 (88%) of initial DF patients showed excellent responses and 2/16 (13%) showed biochemical incomplete responses according to ATA guidelines. A total of 5/7 (%) RAI+ patients showed excellent responses, 1/7 (14%) each showed biochemical incomplete responses and structural incomplete responses. Progression to RAI-R TC occurred in all patients with biochemical (*n* = 3) or structural (*n* = 1) incomplete responses after a mean interval of 29 ± 8 months, and only in 2/21 (10%; *p* = 0.002) of excellent responses after a mean interval of 183.5 ± 5 months. Treatment response could not be evaluated in 4/28 (14%) RAI− patients, of these 2 were lost to follow-up and 2 died within less than a year after initial treatment. All of these presented with iodine-negative lesions at initial imaging. 2 (7%) initially RAI− patients showed excellent responses (following resection of lymph node metastases) and 2 showed biochemical incomplete responses. Twenty-one (75%) of initially RAI− patients showed structural incomplete response.

### 3.5. Survival Analysis

Mean follow-up time after ^18^F-FDG PET was 57.0 ± 43.9 months. Twenty-one patients of this cohort died during follow-up after a median time (range) of 49.7 ± 49.8 months after initial diagnosis. Neoplastic FDG uptake was present in 31 patients. Mean MTV (TLG) in these patients was 58.1 (1159.3) mL. Any measurable MTV or TLG was significantly associated with shorter OS (median OS: 50.2 vs. 133.0 months; *p* < 0.005). Values above the upper quartile for MTV (>49.0 mL) and TLG (>229.0 mL) of our cohort were significantly associated with shorter OS (median OS: 29.0 vs. 56.9 months; *p* < 0.05). Early Tg progression was assessable in 43 patients, and present in 15 (35%), who were significantly associated with shorter OS (57.8 months vs. not reached; *p* < 0.005). As expected, RAI− patients were overrepresented in the cohort of patients with early Tg progression (93% RAI− vs. 7% RAI+ patients).

OS differed significantly depending on AJCC TNM (7th edition) stage (*p* = 0.013) with the shortest median OS being observed in patients with AJCC TNM stage IV (60.3 months vs. 188.2 months in grade III; median OS not reached in stages I and II).

**Figure 3:**
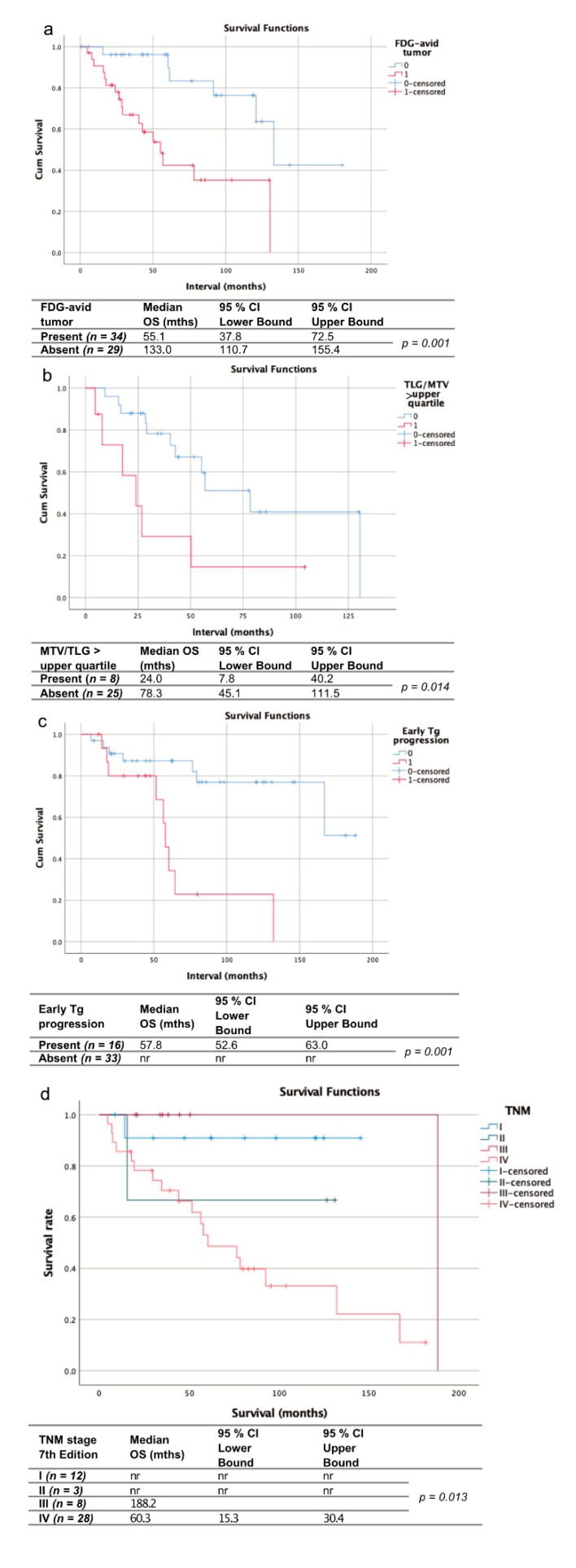
Kaplan-Meier curves and charts showing the overall survival of patients with vs. without ^18^F-FDG-avid tumor at initial FDG-PET (**a**), with metabolic tumor volume/total lesion glycolysis (MTV/TLG) in the upper quartile (**b**) vs. the remaining patients with FDG-avid tumor, patients with early Tg progression vs. those without (**c**) and stratified by the AJCC/TNM stage according to the 7th edition (**d**). nr = not reached

Mean follow-up time after initial diagnosis was 61.1 ± 46.8 months. Sixteen patients of this cohort died during follow-up time, after a median time (range) of 39.0 ± 39.8 (3–160) months (14 RAI−, 1 RAI+). Figure 3 gives an overview of all the assessed parameters.

## 4. Discussion

This study shows a high prevalence of RAI-R disease in patients with PDTC, even at initial diagnosis. 39% of patients without iodine non-avid lesions on initial imaging eventually progress to RAI-R disease, 33% of these progressing within one year. Risk factors for RAI-R PDTC at initial diagnosis or in the course of disease are primary tumor size >40 mm, extrathyroidal extension, and age >55 years. Furthermore, biochemical or structural incomplete response after initial treatment were significantly associated with late (≥12 months) occurrence of RAI-R disease.

The high prevalence of RAI-R disease in PDTC patients with these risk factors calls for the implementation of early cross-sectional imaging for treatment guidance and the evaluation of multimodal treatment. Still, neoplastic RAI uptake was present in 37% of patients and Tg response observed in a considerable fraction of patients. Therefore, PDTC patients should not be automatically precluded from undergoing RAIT.

To the authors’ knowledge, this is the first study to examine the association between RAI-R TC and the aforementioned histopathological or clinical risk factors in PDTC, although prior studies have shown these factors to be generally associated with a poor patient outcome [4,17]. Additionally, a prior study by de la Fouchardiére et al. has shown TERT-mutations to play a significant role in RAI-R PDTC and a higher recurrence rate for PDTC patients with incomplete responses; however, radioiodine-avidity of recurrent lesions was not reported [4].

Our study also shows the potential of TLG and MTV as metrics for poor patient outcome, which is in line with studies on different tumor entities, in which higher MTV and TLG are associated with shorter progression-free survival and overall survival [11,18,19,20,21,22,23,24,25]. Yet, to the authors’ knowledge, this is the first study to specifically analyze PDTC patients. A study by Manohar et al. [11] on RAI-R TC showed a statistically significant association of MTV and TLG > median with progression-free survival and a higher hazard ratio between log-MTV/log-TLG and death. This might be explained by the relatively long overall survival in patients with thyroid cancer. Therefore, depending on the follow-up time a significant association with MTV/TLG might only be observed at the extreme end of the spectrum, which can aide the stratification of patients with a grim vs. intermediate-to-excellent prognosis.

In our study cohort early progression of stimulated Tg after initial RAIT was also significantly associated with shorter OS. Of note, the fraction of RAI-R TC patients among patients without early Tg progression was 30%. A prior study by Wang et al. yielded similar results and shown a significant association between progression of stimulated Tg after the first RAIT on the one hand and the occurrence of RAI-R TC on the other hand [26]. Yet to our knowledge similar analyses have neither been performed in PDTC patients nor with regards to OS. Early Tg progression seems to be of particular value for the stratification of patients with intermediate vs. excellent prognosis. On the other hand, PDTCs can frequently be Tg-negative [27]. Additionally, our cohort patients without a radioiodine avid tumor at initial diagnosis did not undergo a second ^124^I-PET/CT or RAIT and early progression of stimulated Tg was thus not assessable. RAI− were also overrepresented among patients with early Tg progression, thus these differences might just reflect the different outcome between RAI− and RAI+ patients.

Limitations of this study include the small sample size and its retrospective nature. Therefore, the study results need to be confirmed in prospective studies and larger collectives. Additionally, any ETE was classified as such, with no distinction being made between minimal and macroscopic ETE. As most patients were initially diagnosed before the 2017 revision of the AJCC/TNM classification, the degree of ETE was not assessable in multiple cases. However, a series of studies have indeed shown a negative prognostic impact of minimal ETE [28,29]. Another limitation is the potential misclassification of four patients who underwent ^124^I PET but not RAIT as RAI-R TC. As shown by Kist et al. [30], radioiodine-avid lesions can be observed on the WBS after RAIT in patients, in whom ^124^I PET did not reveal any neoplastic iodine-uptake. This was the case in 3/28 patients in our study cohort who underwent both imaging modalities (10.7%), therefore a large impact on the study results seems unlikely.

## 5. Conclusions

Our results confirm a high prevalence of RAI-R TC in PDTC patients with risk factors, such as primary tumor size >40 mm, or extrathyroidal extension, or age >55 years. Patients with these risk factors should be assessed for the presence of iodine negative lesions and evaluated for the need of multimodal treatment, but not automatically precluded from RAIT. Additionally, early Tg progression, MTV, and TLG appear to be promising metrics to stratify patient prognosis, and subsequently aide treatment planning.

## Figures and Tables

**Table 1:** Patient characteristics for the assessment of radioiodine refractory (RAI-R) poorly differentiated thyroid carcinoma (PDTC) at initial disease and the prognostic factor of early thyroglobulin (Tg) progression.

Age	Mean (Range)	58.5 (15–87)
	>55 years, *n* (%)	30 (59)
Sex	Male, *n* (%)	23 (45)
	Female, *n* (%)	28 (55)
Size of primary	>40 mm, *n* (%)	31 (61)
	≤40 mm, *n* (%)	15 (29)
	Tx, *n* (%)	2 (4)
	n/a, *n* (%)	3 (6)
ETE	Present, *n* (%)	28 (55)
	Absent, *n* (%)	21 (41)
	Tx, *n* (%)	2 (4)
Stage	N0M0, *n* (%)	19 (37)
	N1M0, *n* (%)	10 (20)
	N0M1, *n* (%)	9 (18)
	N1M1, *n* (%)	13 (25)
Tg	Initial, mean (range)	1734.6 (0–49,362)
	Early Tg progression n/a, *n* (%)	8 (16)
	Early Tg progression, *n* (%)	15 (29)
	No early Tg progression, *n* (%)	28 (55)
	Med. OS, early Tg progression, months	57.8
	Med. OS, no early Tg progression, months	nr
Category	RAI-R TC, *n* (%)	28 (55)
	Radioavid TC, *n* (%)	7 (14)
	Disease-free, *n* (%)	16 (31)
Progression to RAI-R TC	At initial diagnosis, *n* (%)	25 (49)
	≤12 months, *n* (%)	3 (6)
	>12 months, *n* (%)	6 (12)
	Not observed, *n* (%)	17 (33)
AJCC TNM stage	I	12 (24)
	II	3 (6)
	III	8 (16)
	IV	28 (55)

PDTC: Poorly differentiated thyroid carcinoma, n/a: not available, med.: median, OS: overall survival, nr: not reached, RAI-R TC: Radioiodine refractory thyroid cancer.

**Table 2:** Patient characteristics for the assessment of positron-emission tomography (PET) parameters as prognostic factors for overall survival.

Age	Mean (Range)	58.5 (13–87)
Sex	Male, *n* (%)	24 (43)
	Female, *n* (%)	32 (57)
Stage	N0M0, *n* (%)	25 (45)
	N1M0, *n* (%)	8 (14)
	N0M1, *n* (%)	11 (20)
	N1M1, *n* (%)	12 (21)
FDG-avid tumor	Present, *n* (%)	31 (55)
	Absent, *n* (%)	25 (45)
	Med. Survival FDG-avid tumor present, months	50.2
	Med. Survival FDG-avid tumor absent, months	133.0
MTV	Mean (range), mL	58.1 (0.2–468.9)
	Upper quartile, mL	229.0
	Med. Survival MTV > upper quartile, months	29.0
	Med. Survival MTV < upper quartile, months	56.9
TLG	Mean (range), mL	1159.3 (2.3–15,175.7)
	Upper quartile, mL	49.0
	Med. Survival MTV > upper quartile, months	29.0
	Med. Survival MTV < upper quartile, months	56.9
Follow-up	Overall survival, mean ± SD	57.0 ± 43.9

PDTC: poorly differentiated thyroid carcinoma, MTV: metabolic tumor volume, TLG: total lesion glycolysis, SD: Standard deviation.

**Table 3:** Risk factors for radioiodine refractory disease at initial diagnosis and in the course of disease.

RAI-R TC Occurrence	ETE	No ETE	Significance
Initial RAI-R TC	75.0%	28.6%	*p* = 0.001
Ever RAI-R TC	89.3%	33.3%	*p* < 0.001
	**Primary > 40 mm**	**Primary ≤ 40 mm**	**Significance**
Initial RAI-R TC	64.5%	26.7%	*p* = 0.017
Ever RAI-R TC	80.6%	26.7%	*p* = 0.001
	**Age > 55**	**Age ≤ 55**	**Significance**
Initial RAI-R TC	66.7%	38.1%	*p* = 0.041
Ever RAI-R TC	83.3%	42.9%	*p* = 0.003

ETE: extrathyroidal extension, RAI-R TC: radioiodine refractory thyroid cancer.

**Table 4:** (**a**) Multivariate model of risk factors for RAI-R TC at initial diagnosis. ETE = extrathyroidal extension; CI = confidential interval. (**b**) Multivariate model of risk factors for RAI-R TC during the course of disease. ETE = extrathyroidal extension; CI = confidential interval.

**(a)**				
**Risk factors**	***p***	**Hazard Ratio**	**95% CI for Hazard Ratio**	
			**CI Lower**	**CI Upper**
ETE	0.016	5.171	1.354	19.747
Primary size >40 mm	0.081	3.669	0.85	15.838
**(b)**				
	***p***	**Hazard Ratio**	**95% CI for Hazard Ratio**	
			**CI Lower**	**CI Upper**
ETE	0.003	14.821	2.526	86.943
Primary size >40 mm	0.007	11.596	1.932	69.612

## Data Availability

The datasets generated and/or analysed during the current study are not publicly available due to privacy laws but are available from the corresponding author on reasonable request.
